# Simplicity within complexity: Seasonality and predictability of hospital admissions in the province of Ontario 1988–2001, a population-based analysis

**DOI:** 10.1186/1472-6963-5-13

**Published:** 2005-02-04

**Authors:** Ross EG Upshur, Rahim Moineddin, Eric Crighton, Lori Kiefer, Muhammad Mamdani

**Affiliations:** 1 Department of Family and Community Medicine, University of Toronto, 263 McCaul Street, Toronto, ON M5T 1W7, Canada; 2 Department of Public Health Sciences, University of Toronto, McMurrich Building, 12 Queen's Park Crescent W., Toronto, ON M5S 1A8, Canada; 3 Primary Care Research Unit, Sunnybrook and Women's College Health Sciences Centre, 2075 Bayview Ave., #E-349, Toronto, ON M4N 3M5, Canada; 4 Institute of Clinical Evaluative Sciences, 2075 Bayview Avenue, Toronto, ON M4N 3M5, Canada; 5 Faculty of Pharmacy, University of Toronto, 19 Russell Street, Toronto, ON M5S 2S2, Canada

## Abstract

**Background:**

Seasonality is a common feature of communicable diseases. Less well understood is whether seasonal patterns occur for non-communicable diseases. The overall effect of seasonal fluctuations on hospital admissions has not been systematically evaluated.

**Methods:**

This study employed time series methods on a population based retrospective cohort of for the fifty two most common causes of hospital admissions in the province of Ontario from 1988–2001. Seasonal patterns were assessed by spectral analysis and autoregressive methods. Predictive models were fit with regression techniques.

**Results:**

The results show that 33 of the 52 most common admission diagnoses are moderately or strongly seasonal in occurrence; 96.5% of the predicted values were within the 95% confidence interval, with 37 series having all values within the 95% confidence interval.

**Conclusion:**

The study shows that hospital admissions have systematic patterns that can be understood and predicted with reasonable accuracy. These findings have implications for understanding disease etiology and health care policy and planning.

## Background

Health care is a complex human endeavor constituted by the interaction of multiple professions, organizations, industries, technologies and the public. Health itself is also a complex concept, with multiple determinants including genetic, socio-cultural, economic and environmental influences [1]. At the centre of this complex system is the hospital. Arguably, after a physician visit, the hospital admission represents the key event in the delivery of health care.

Do hospital admissions have consistent patterns? While individual diseases are extensively studied, there is a paucity of systematic approaches to the study of health care events. Epidemiology is not regarded as a science with the predictive accuracy and explanatory power of the physical sciences [2]. Health services research is in its scientific infancy and is directed towards policy and practice, however, recent trends in theoretical epidemiology have focused on more powerful computational approaches [3].

Using time series analysis, our research program investigates seasonality in the occurrence of health care events. Seasonality is an important aspect of disease manifestation as well as a clue to the etiology of disease. Our initial studies explored seasonality in hospital admissions in discrete disease categories including asthma [4], falls [5] and aortic aneurysms [6]. Subsequently, we hypothesized and confirmed that the hospital admissions in the system considered in totality also demonstrated consistent seasonal effects [7].

Consistent seasonal behavior suggests the possibility of predictable behavior. To the best of our knowledge, there are no studies systematically evaluating the seasonality and predictability of multiple hospital admissions using health services data. We therefore assessed the seasonality and predictability of the most common causes of hospital admission in the province of Ontario, Canada.

## Methods

We conducted a retrospective, population-based study to assess temporal patterns in hospitalisations for the 52 most common admission discharge diagnoses from April 1, 1988 to December 2001. Approximately 14 million residents of Ontario eligible for universal healthcare coverage during this time were included for analysis. The Canadian Institute for Health Information Discharge Abstract Database was used to obtain information on the most responsible diagnosis. This database records discharges from all Ontario acute care hospitals, documenting a scrambled patient identifier, date of admission and discharge, up to 16 diagnoses as coded by the International Classification of Diseases, Ninth Revision, Clinical Modification (ICD-9-CM), and up to 10 procedures.

Researchers using these databases have found that diagnoses and surgical procedures are coded with a high degree of accuracy. There is very little missing information in the Ontario databases; other studies have similarly found that less than 1 percent of the basic information on patients is missing in various provincial databases [8-10].

The 52 most common discharges diagnoses over the 10 years were identified by summing all admissions and calculating in rank order the frequencies of admission. Owing to the influence of obstetric related admissions, we limited obstetric codes to the consideration of singleton births. Categories of closely related health conditions (such as myocardial infarction) were combined.

Numerator data consisted of the total number of discharges for each month for each of the most responsible diagnoses. Denominator data was derived from annual census data for each age group for residents of Ontario provided by Statistics Canada. Monthly population estimates were derived through linear interpolation. All transfers from within one acute care hospital to another within this study group were excluded from the analysis. To take into account the population changes over time we analyzed monthly admission rates per 100,000.

### Analytic method

This study employed time series methods to assess the presence of statistically significant seasonality, the strength of the seasonal effect and the predictability of the time series. A time series can be decomposed as the sum or product of trend, seasonality, and random components. Trend is the long term movement of the series which is a systematic component that changes over time and generally does not repeat itself within the time range of the available data. If we eliminate the trend then the time series will consist of seasonal and random components.

### Assessment of seasonality

Analysis of the data involved the use of the following statistical techniques in identical fashion to each series in order to assess statistical significance of seasonal patterns and the consistency and magnitude of seasonal effect. Spectral analyses were conducted to detect statistically significant seasonality. Spectral analysis detects periodicity in time series, by plotting the periodogram or spectral density of the series against the period or frequency [11]. The data series was de-trended using moving averages prior to conducting spectral analysis. Two tests for the null hypothesis that the series is strictly white noise were conducted. The Fisher Kappa (FK) Test is designed to detect one major sinusoidal component buried in white noise, whereas the Bartlett Kolmogorov Smirnov (BKS) Test accumulates departures from the white noise hypothesis over all frequencies [12]. Finally, R-squared autoregression coefficients (R^2^_Autoreg_) were calculated. Autoregression uses the coefficient of determination of the autoregressive regression model fitted to the data, and can be used for quantifying the strength of the seasonality within a set of serially correlated observations as occurs with time series data [13]. The R^2^_Autoreg _is interpreted the same way as the coefficient of determination in classic regression: values from 0 to less than 0.4 represent non-existent to weak seasonality, 0.4 to less than 0.7 moderate to strong seasonality, and 0.7 to 1 strong to perfect seasonality. The magnitude of the R^2^_Autoreg _shows how well the next value can be predicted when the seasonal component is the only predictor. In other words it shows the contribution of seasonality in the total variation of the data. Thus 1-R^2^_Autoreg _would be the variance that remains unexplained [13]. When the autoregression procedure is applied to observed data, it is important to validate the stationarity of the series as the R^2^_Autoreg _may be underestimated when the seasonal variation is non-stable. To account for this, data transformations were conducted where appropriate, to stabilize the seasonal variations [13]. All statistical analyses were performed using SAS (v8.2).

### Predictive modeling

Of the 160 monthly observations for each series, the first 148 (April 1988 to December 2000) were used for fitting the model and estimating the parameters. We set aside the last 12 observations (January to December 2001) for assessing the performance of the suggested model and used the rest for fitting the model and estimating the parameters. We applied the first order differencing to eliminate the trend [14] and then used a very simple regression model to predict 12 new monthly observations for each series. We compared the observed 12 observations with the corresponding predicted values. Then we checked to see which observed value falls outside the 95 percent confidence interval.

Suppose *n *monthly observations *x*_1_, *x*_2_, ..., *x*_*n *_are available and we are interested in predicting the next *k *unobserved data points *x*_*x*+1_, *x*_*x*+2_,..., *x*_*x*+*k *_using the *n *observed data points. Here we will assume that the time series is an additive composition of trend, seasonality, and random components. The multiplicative case can be converted to additive by simply taking the log transformation. The time plot of the series did not indicate large changes in the variations of the amplitude of either seasonal or irregular components of the series whereas the level of the trend increased or decreased. Thus an additive model is appropriate. The first component we should deal with is trend. Visual inspection of the time plots of the 52 series indicate different trend patterns ranging from simple linear to more complex nonlinear patterns. We did not attempt to model the trend component parametrically as estimating the pattern of the trend components globally by a closed mathematical function of time may severely misestimate the true trend beyond the range of fitting period. Instead we decided to use the first order differencing to eliminate the trend component. The first order differencing of a time series *x*_*t*_, *t *= 1,2, ..., *n *is the series *w*_*t*_, *t *= 2,3, ..., *n *where *w*_*t *_= *x*_*t *_- *x*_*t*-1 _[14]. Visual inspection of the time plots of the differenced series showed elimination of the trend components. For monthly rates of hospitalization data it is reasonable to anticipate seasonal components of order 12 and 6 due to seasonal variation of the weather or administration (e.g. winter, Christmas, and vacation season). This was confirmed in spectral analysis. By modifying the components of the following regression equation we can model the series at different seasonal orders.

In the regression model we included  for seasonal factors of period 12 and 6. Thus the regression model takes the following form



where *β*_*i*_'*s *can be estimated through linear regression framework. Having fitted the model, one can substitute *t *= *n *+ 1, *n *+ 2, ..., *n *+ *k *to estimate the next *k *differenced observations with their corresponding confidence intervals. The predicted differenced data points can be converted to raw data points by applying the following simple transformation:

*x*_*n*+*j *_= *w*_*n*+*j *_+ *x*_*n*+*j*-1_, *j *= 1,2, ..., *k*

Confidence intervals can be transferred in a similar manner. For *j *> 1we can substitute the predicted values for *x*_*n*+*j*-1_.

## Results

**Figure 1:**
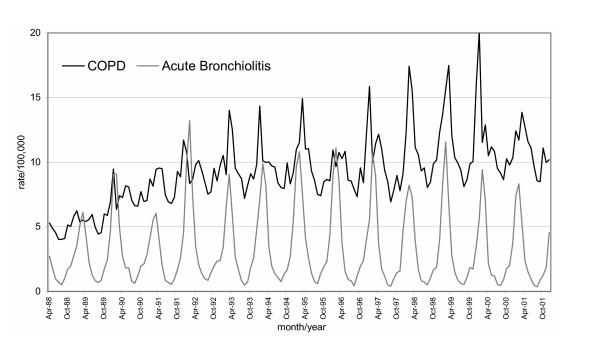
Time plots (rates per 100,000 population) of highly seasonal hospital admission patterns: Chronic obstructive pulmonary disease and bronchiolitis.

**Figure 2:**
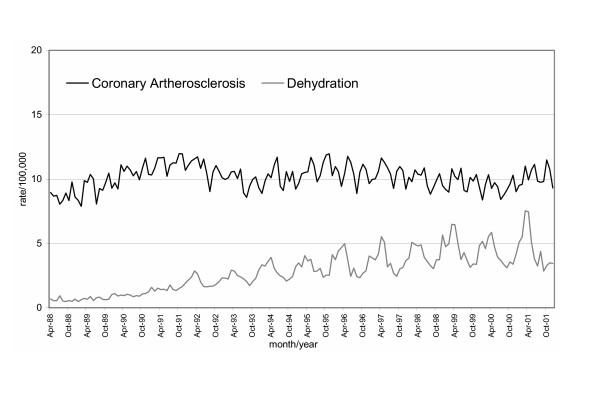
Time plots (rates per 100,000 population) of moderately seasonal and non-linear trend in hospital admission patterns: Coronary atherosclerosis and dehydration.

A total of 6,560,210 million admissions were included in the analysis. Figures 1 and 2 provide examples of the heterogeneity of the time series. There is visual evidence of non-linearity and clear seasonality in the time plot graphs.

Table 1 provides the Fisher Kappa and BKS and R^2^_Autoreg _test statistics for each diagnosis, rank ordered by R^2^_Autoreg, _and the number of predictions that fall outside the 95 percent confidence interval. The R^2^_Autoreg _values range from a high of 0.95 (bronchiolitis) to a low of 0.11 (infantile cataract). Fourteen series showed evidence of strong seasonality (R^2^_Autoreg _greater than 0.7), nineteen series showed evidence of moderate seasonality (R^2^_Autoreg _between 0.4 and 0.69) and eleven showed evidence of weak seasonality (R^2^_Autoreg _less than 0.4). Time series with strong seasonal effects by R^2^_Autoreg _also showed consistent statistical evidence of seasonality by BKS and Fisher Kappa tests. Those with moderate and weak evidence of seasonality by R^2^_Autoreg _showed inconsistent statistical evidence of seasonality by BKS and Fisher Kappa tests.

**Table 1:** Statistical summary of seasonality and predictability of the 52 admission time series

**Health Outcome**	**R^2^_Autoreg_**	**Fisher Kappa (p-value)**^1^	**BKS (p-value)**^1^	**# outside 95% CI**^2^
**Acute bronchiolitis**	**0.95**	**76.56 (<0.01)**	**0.77 (<0.01)**	**0**
Non-infectious gastroenteritis	0.91	66.28 (<0.01)	0.65 (<0.01)	0
**Pneumonia/influenza**	**0.88**	**68.64 (<0.01)**	**0.68 (<0.01)**	**0**
Osteoarthritis	0.86	49.81(<0.01)	0.37 (<0.01)	0
**Appendicitis**	**0.84**	**52.99 (<0.01)**	**0.50 (<0.01)**	**0**
Uterine fibroids	0.83	40.05 (<0.01)	0.27 (<0.01)	0
**Congestive heart failure**	**0.82**	**44.14 (<0.01)**	**0.42 (<0.01)**	**0**
Previous C-section	0.82	44.52 (<0.01)	0.39 (<0.01)	0
**Prostatic hyperplasia**	**0.80**	**36.49 (<0.01)**	**0.31 (<0.01)**	**0**
Singleton birth	0.76	39.20 (<0.01)	0.37 (<0.01)	0
**Croup**	**0.75**	**47.84 (<0.01)**	**0.56 (<0.01)**	**0**
Diverticulosis	0.75	29.57 (<0.01)	0.33 (<0.01)	0
**Excessive menstruation**	**0.72**	**34.02 (<0.01)**	**0.26 (<0.01)**	**0**
Chronic obstructive pulmonary disease	0.71	50.14 (<0.01)	0.50 (<0.01)	0
**Urinary tract infection**	**0.69**	**52.24 (<0.01)**	**0.48 (<0.01)**	**3**
Coronary atherosclerosis	0.69	31.60 (<0.01)	0.21 (<0.01)	0
**Kidney stones**	**0.67**	**40.21 (<0.01)**	**0.35 (<0.01)**	**0**
Breast cancer	0.67	39.47 (<0.01)	0.24 (<0.01)	0
**MyocardiaI infarction**	**0.67**	**32.48 (<0.01)**	**0.30 (<0.01)**	**1**
Gall bladder	0.66	34.69 (<0.01)	0.27 (<0.01)	0
**Prostate cancer**	**0.62**	**33.42 (<0.01)**	**0.26 (<0.01)**	**3**
Senile cataract and cataract unspecified	0.60	26.09 (<0.01)	0.27 (<0.01)	0
**Acute pancreatitis**	**0.60**	**25.30 (<0.01)**	**0.18 (<0.05)**	**0**
Threatened premature labour	0.59	26.74 (<0.01)	0.19 (<0.01)	1
**Gall bladder w/acute cholecystitis**	**0.57**	**20.08 (<0.01)**	**0.15 (NS)**	**0**
Convulsions	0.54	22.46 (<0.01)	0.21 (<0.01)	0
**Trochanteric fracture**	**0.53**	**22.62 (<0.01)**	**0.14 (NS)**	**0**
Chronic tonsillitis	0.51	20.82 (<0.01)	0.24 (<0.01)	0
**Recurrent manic depression (depressed phase)**	**0.51**	**25.43 (<0.01)**	**0.20 (<0.01)**	**0**
Premature rupture of membrane	0.50	32.01 (<0.01)	0.25 (<0.01)	1
**Displacement of inter-lumbar disc**	**0.50**	**26.38 (<0.01)**	**0.18 (<0.01)**	**1**
Dehydration	0.50	55.40 (<0.01)	0.58 (<0.01)	2
**Syncope and collapse**	**0.48**	**22.57 (<0.01)**	**0.18 (<0.05)**	**5**
Uncomplicated diabetes	0.48	22.54 (<0.01)	0.23 (<0.01)	0
**Lung cancer**	**0.46**	**19.41 (<0.01)**	**0.12 (NS)**	**0**
Depressive disorder	0.45	12.28 (<0.01)	0.14 (NS)	1
**Fractured femur**	**0.44**	**12.72 (<0.01)**	**0.10 (NS)**	**3**
Unilateral inguinal hernia	0.43	16.52 (<0.01)	0.18 (<0.01)	0
**Abdominal pain**	**0.43**	**19.15 (<0.01)**	**0.26 (<0.01)**	**0**
Transient cerebral ischemia	0.41	18.42 (<0.01)	0.12 (NS)	2
**Acute but ill defined cardiovascular disease**	**0.40**	**14.69 (<0.01)**	**0.19 (NS)**	**0**
Angina	0.40	11.72 (<0.01)	0.14 (NS)	0
Unspecified intestinal obstruction	0.38	10.80 (<0.01)	0.15 (<0.05)	0
**Other acute ischaemic heart disease**	**0.36**	**17.08 (<0.01)**	**0.15 (<0.05)**	**1**
Recurrent manic depression (manic phase)	0.35	13.77 (<0.01)	0.10 (NS)	4
**Fetal distress**	**0.34**	**20.24 (<0.01)**	**0.26 (<0.01)**	**0**
Spontaneous abortion unspecified	0.33	10.95 (<0.01)	0.12 (NS)	0
**Stroke**	**0.31**	**10.32 (<0.01)**	**0.14 (NS)**	**0**
Chest pain (nonspecific)	0.29	11.34 (<0.01)	0.14 (NS)	4
**Gastrointestinal bleed**	**0.26**	**7.84 (<0.05)**	**0.14 (NS)**	**0**
Other IHD	0.17	6.24 (NS)	0.12 (NS)	0
**Infantile cataract**	**0.11**	**4.85 (NS)**	**0.28 (<0.01)**	**0**

^1 ^NS = not significant (p > 0.05)

^2 ^95% CI = 95% confidence interval

In total, 96.5 percent of the predictions fell within the 95 percent confidence interval (602/624). In terms of complete series, the performance of the proposed predictive model is very good. Overall 37 (37/52 = 73 percent) had all 12 observed values falling within 95 percent prediction intervals, 10 series had only 1 observed value outside prediction limits and 4 series had 2 observed values outside 95 percent prediction intervals. For the worst case, only 1 series had 4 out of 12 observed values falling outside the 95% prediction intervals. The standard deviations for the confidence intervals of the predicted values are within 2 admissions per 100,000 for 48 of the 52 series (data not shown).

## Discussion

Hospital admissions in the province of Ontario show remarkable consistency and predictability of occurrence. A heterogeneous group of health conditions are represented in the sample including surgical and medical conditions, acute and chronic diseases, communicable and non-communicable diseases. The performance of the proposed model for predicting the one-year ahead number of hospital admissions in the province of Ontario is excellent for the 52 most frequent hospital admissions series considered in this study.

Are these results of significance? We believe so. Most health care planning is based on what could be termed the 'invariance principle' that holds that all events are equally likely to happen and therefore hospitals should be staffed and managed accordingly [15]. Our study indicates that demand for hospital services varies, can be predicted with a high degree of accuracy and therefore planning and resource allocation could possibly be reorganized to reflect this knowledge. Furthermore, there are significant seasonal fluctuations to at least one third of the series analyzed, indicating that planning could be tailored to predictable demands. Understanding such seasonal patterns also promises to shed light on disease causality as not all highly seasonal conditions can be explained by infectious diseases known to have seasonal occurrence.

Our study is limited to the context of Ontario, and is applicable at a population level. Focusing on the most responsible diagnosis may bias the account of seasonal occurrence, although this bias is likely to be non-differential. In this study we focused on total counts for each most responsible diagnosis, which may obscure significant variation in rates between age and gender.

The proposed methods enjoy simplicity and stability. The prediction approach does not require model selection or any other sophisticated statistical methods. Selecting an appropriate seasonal model can be a challenging task in time series analysis. For example, the Box Jenkins approach is popular for selecting linear time series models. In this approach sometimes the analyst has to select a model subjectively from among several potentially appropriate models. Our proposed regression model does not require model selection.

The first order differencing eliminates trend; sin and cosine terms estimate the seasonal factors. The simple regression model works well for highly seasonal to non-seasonal data. Although the seasonal factors of some of the series are changing over time, the simple first order differencing in conjunction with the regression model forecast the future observations within the 95 percent confidence bounds. The confidence intervals around the predicted values are tight, reflecting the accuracy of the projections. This attenuates concerns expressed about the robustness of predictive models in epidemiology [16].

## Conclusion

The results of this study demonstrate a simplicity underlying the complexity of hospital admissions. We believe these results are promising and can lead to more rational planning of hospital resources and open up areas of exploration for understanding the determinants of disease causation, specifically in those conditions with moderate to strong seasonality. Further research is necessary to look at whether more complex models have greater predictive power, and whether the analytic approach is robust at different time and space aggregations.

## Competing interests

The author(s) declare that they have no competing interests.

## Authors' contributions

RU conceived the study and wrote the first draft. RM contributed the statistical analysis. MM, LK and EC made substantial contributions to the design and interpretation of the data. All authors contributed to subsequent drafts, have read and approve of the content of the final submitted manuscript.

All authors have access to all data in the study and they hold final responsibility for the decision to submit for publication.

## Pre-publication history

The pre-publication history for this paper can be accessed here:


